# Focussing Protons from a Kilojoule Laser for Intense Beam Heating using Proximal Target Structures

**DOI:** 10.1038/s41598-020-65554-4

**Published:** 2020-06-10

**Authors:** C. McGuffey, J. Kim, M. S. Wei, P. M. Nilson, S. N. Chen, J. Fuchs, P. Fitzsimmons, M. E. Foord, D. Mariscal, H. S. McLean, P. K. Patel, R. B. Stephens, F. N. Beg

**Affiliations:** 1 Center for Energy Research, University of California, San Diego, La Jolla, CA 92093-0417 USA; 2 General Atomics, P.O. Box 85608, San Diego, CA 92186-5608 USA; 3 Laboratory for Laser Energetics, University of Rochester, Rochester, NY 14623 USA; 4 Laboratoire pour l’Utilisation des Lasers Intenses - CNRS, CEA, UPMC Univ Paris 06: Sorbonne Université, Ecole Polytechnique, Institut Polytechnique de Paris, F-91128 Palaiseau cedex, France; 5 Lawrence Livermore National Laboratory, P.O. Box 808, Livermore, CA 94550 USA; 6 Extreme Light Infrastructure - Nuclear Physics/Horia Hulubei National Institute for R&D in Physics and Nuclear Engineering, Bucharest-Magurele, 077125 Romania

**Keywords:** Nuclear fusion and fission, Plasma-based accelerators

## Abstract

Proton beams driven by chirped pulse amplified lasers have multi-picosecond duration and can isochorically and volumetrically heat material samples, potentially providing an approach for creating samples of warm dense matter with conditions not present on Earth. Envisioned on a larger scale, they could heat fusion fuel to achieve ignition. We have shown in an experiment that a kilojoule-class, multi-picosecond short pulse laser is particularly effective for heating materials. The proton beam can be focussed via target design to achieve exceptionally high flux, important for the applications mentioned. The laser irradiated spherically curved diamond-like-carbon targets with intensity 4 × 10^18^ *W*/*cm*^2^, producing proton beams with 3 *MeV* slope temperature. A Cu witness foil was positioned behind the curved target, and the gap between was either empty or spanned with a structure. With a structured target, the total emission of Cu K*α* fluorescence was increased 18 fold and the emission profile was consistent with a tightly focussed beam. Transverse proton radiography probed the target with ps order temporal and 10 *μm* spatial resolution, revealing the fast-acting focussing electric field. Complementary particle-in-cell simulations show how the structures funnel protons to the tight focus. The beam of protons and neutralizing electrons induce the bright K*α* emission observed and heat the Cu to 100 *eV*.

## Introduction

High-intensity proton beams generated by ultrashort pulse laser-matter interactions^1–3^ were immediately recognized as a powerful tool for the creation of Warm Dense Matter (WDM)^4,5^. These beams have since found widespread use in High Energy-Density physics studies as isochoric heaters that allow study of conditions similar to those in the interior of planets^6^, as probes of complex objects^7^ and of transient electric and magnetic fields^8–11^ with micron scale resolution^12^, or for inducing nuclear reactions to create directional neutron beams^13,14^. These intense proton beams also hold promise as the ignitor in Fast Ignition (FI) fusion^15–17^ if the total beam energy can be scaled up.

The utility of these beams comes from their high particle energy (10’s of MeV) and energy bandwidth, low source emittance^18^, and short (multi-ps) initial pulse duration produced by the target normal sheath acceleration (TNSA) mechanism^3^. The beams initially have a pulse duration similar to the laser duration down to a few picoseconds. A bunch length can then be estimated for different source-to-sample distances by considering the velocity dispersion of the broadband beam. While advanced acceleration mechanisms are predicted to produce very high energy protons and ions^19–22^, the well-established TNSA scheme has a distinct advantage in that the beams are very easy to produce and simply curving the target can focus the beam. TNSA-hybrid mechanisms hold the current record for highest energy, up to 100 *MeV*^23,24^.

Focussing the ion beam increases the flux and widens the possibilities for all the above-listed applications. The extremely low beam transverse emittance of TNSA allows a curved hemisphere target to focus the proton beam to small spot size (50–100 *μm*), as demonstrated on small (10 *J*, 100 *fs*^4^, 75 *J*, 700 *fs*^25^) and medium scale facilities (400 *J*, 400 *fs*^26^, 170 *J*, 700 *fs*^5^). However, the ultimate achievable focussing is limited by hot electron pressure in the radially shrinking proton beam itself^27^. The consequence of this is that for high charge, tightly focussed beams, the protons do not move ballistically through a focus, and a spherically curved target is not enough, by itself, to achieve optimal focussing^28^. Early experimental data on the kiloJoule OMEGA EP laser showed that proton beam focussing from free-standing curved targets was indeed limited by this non-ballistic behavior at this higher energy laser drive^29^.

However, the paper by Bartal et al. also discovered that by attaching a cylindrical or conical structure to the target, the beam could again be tightly focussed. Simulations showed that this was caused by a focussing electrostatic field that rapidly-grew on the inside of the structure as hot electrons from the interaction escaped the target and populated a sheath along the structure walls^30,31^. Related field effects have been studied in detail for periodic target structures with a $\tau =30\,fs$ laser^32^. The finding that the cone focussed the beam was particularly appealing for FI, which is often envisioned with a cone embedded in a spherical target. It is also broadly appealing for high beam current applications to overcome the beam pressure limitation because focussing caused by the cone can be expected to be more significant as the laser energy and pulse duration are increased, motivating the experiment presented here.

In this work, we show a highly effective method for focussing protons and heating a secondary target using conical-shaped target structures with fundamentally different laser conditions than previous proton heating experiments. The high energy and pulse duration of the OMEGA EP drive laser produced a proton beam that, when focussed, achieved exceptionally high peak beam density. This dramatically increases the prospects for using protons for isochoric heating and the intensity-hungry applications mentioned above. This is an important demonstration that a structure behind the target is still effective for focussing a proton beam from a much higher energy and longer duration laser than the Bartal result. Furthermore, the OMEGA EP orthogonal short pulse beam was used to drive a second beam of protons which radiographed the fields responsible for focussing. In the second half of the paper, we present particle in cell simulations that confirm the focussing and show the mechanism in detail. In two-step simulations, the heating of a secondary target is evaluated, with an expected temperature of $\mathrm{100\ }eV$. To conclude, we show how a steeper cone geometry may increase heating and constrict the proton beam to the size required for proton FI.

## Findings

**Figure 1:**
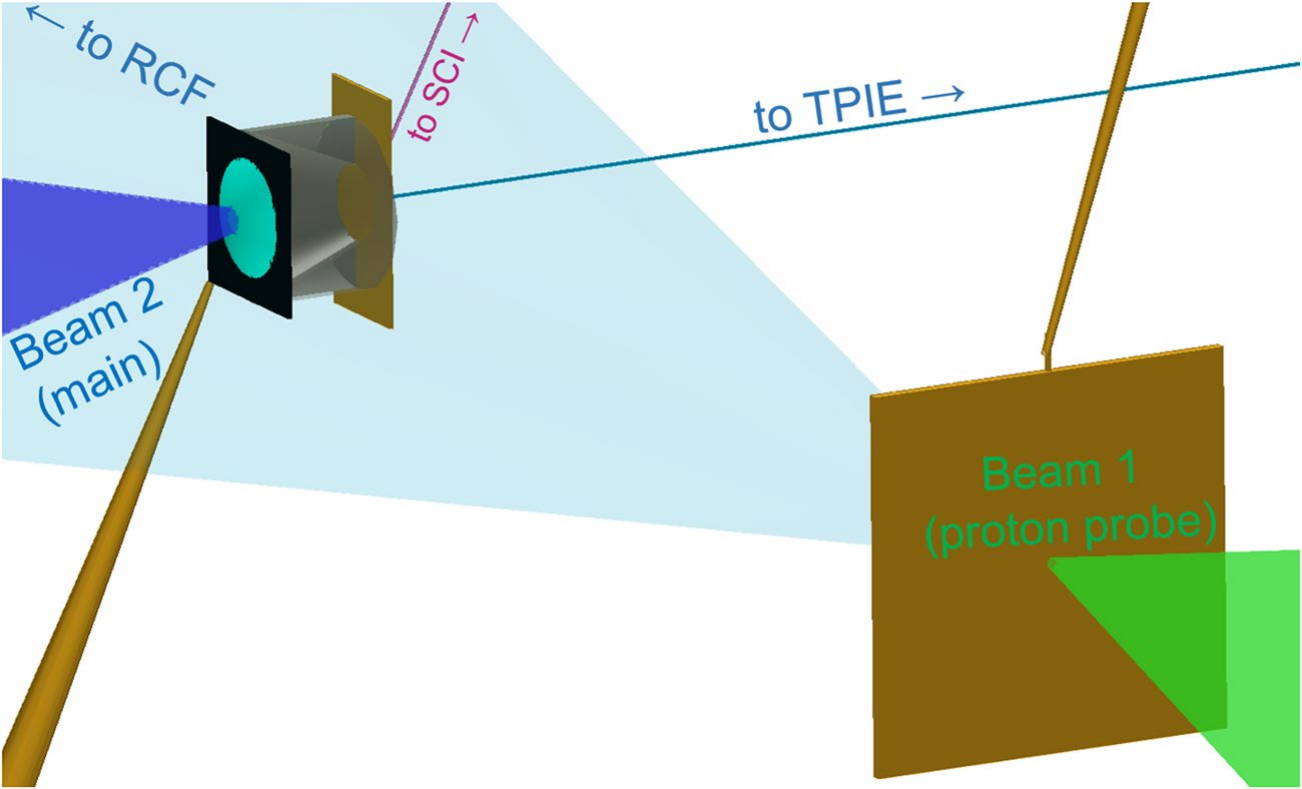
Experimental configuration drawn with VISRAD software. OMEGA EP short pulse main beam irradiated a curved diamond-like carbon target attached to an Al cone to produce an ultrahigh intensity proton beam, directed into various transport media. The cone is shown cutaway. A Cu diagnostic layer was glued to the back of the target. The other short pulse beam produced a transverse proton probing beam shown as a transparent cone pointing toward the RCF stack. Lines of sight toward TPIE and the SCI x-ray imager as shown as thin lines. Stalks supporting each target are shown as brown, tapered lines. For the freestanding case, a third stalk supported the Cu foil.

Proton focussing measurements were taken for the first time in the kiloJoule regime at the OMEGA EP facility using a $1.25\,kJ,\,10\,ps$ short pulse laser along with proton spectrum measurements using a Thomson parabola diagnostic named Thomson Parabola Ion Energy Analyzer (TPIE). The laser was incident on a diamond-like carbon spherical cap (“hemi”). Protons were focussed into metallic foils and their transmitted spectrum was recorded. The flux was sufficient to induce bright K*α* x-rays from the rear Cu layer as measured by a single-hit spectrometer and a Spherical Crystal Imager (SCI). A second, orthogonal OMEGA EP beam irradiated flat foils with 850 *J* in 10 *ps* in order to probe the interaction with broad-energy protons of order *MeV*. Due to the protons’ velocity dispersion, different energy protons probed the interaction plane at different delays. They were then discriminated by energy using a stack of RadioChromic Film (RCF) detectors. The different layers of film, or ‘frames’, resolve the target dynamics as the laser arrives with resolution of a few ps. Further details about the laser, target, and diagnostics are given in the Methods Section while the experimental configuration is illustrated in Fig. 1.

**Figure 2:**
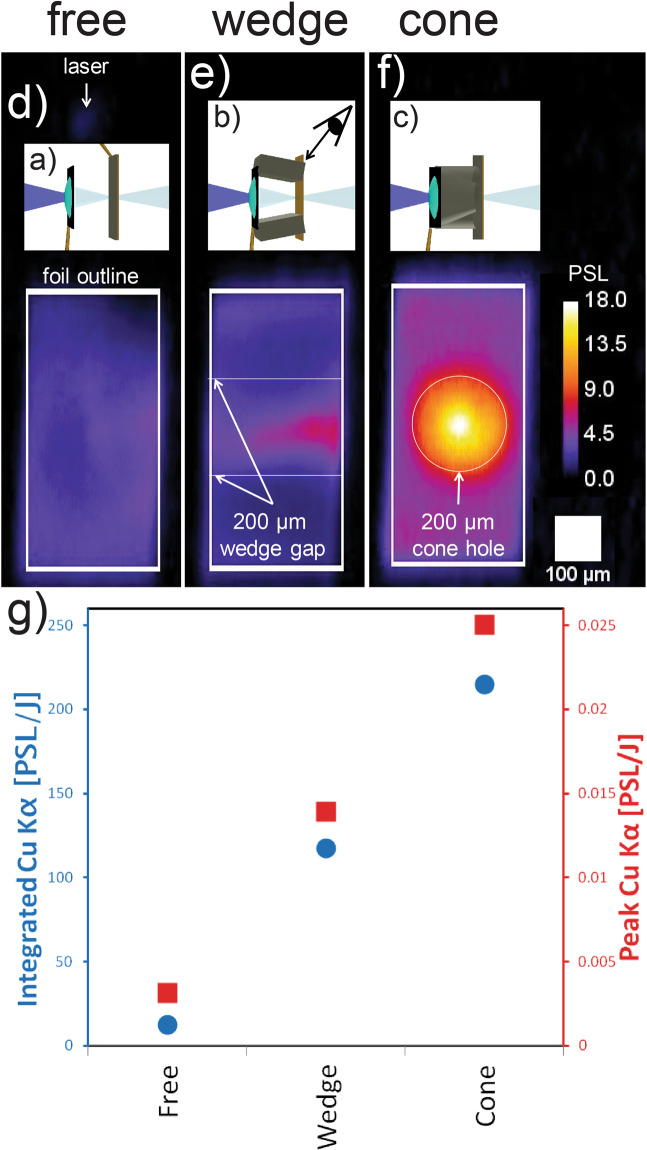
Three target types (insets (**a**–**c**) drawn with VISRAD software) used in the OMEGA EP experiment, and their corresponding emission profiles of 8.048 *KeV* Cu K*α* (**d**–**f**). (**g**) Plot of the emission brightness from the foil center (peak pixel) and integrated over the full foil dimension. The unit PSL stands for Photo-Stimulated Luminescence, the standard measure of the deposited energy in the imaging plate detector. The SCI viewing position for all cases is illustrated in (**b**). For orientation, note the dim signal in (**d**) (above inset (**a**)) which is due to hemi self-emission and indicates the laser interaction point.

The SCI data are shown in Fig. 2 for the three target types. For the case of freestanding, separated foils, illustrated in Fig. 2(a), the Cu K*α* signal was weak and diffuse over the entire Cu foil, Fig. 2(d). This confirmed that the inherently diverging electron beam from the interaction contributed minimally to the Cu K*α* signal at this standoff distance. Signal was darker in the top-right corner, corresponding to the stalk connection point, visible in Fig. 2(a). In contrast to this diffuse signal, for the case in which the gap was bridged by a wedged structure, Fig. 2(b), signal was increased on the wedge center plane by 5× as seen in Fig. 2(e). The emission is greatest in the center, not at the edges of the wedge connection vertices, and no signal enhancement is observed in the region directly in contact with the wedges. This suggests that the signal is due to freestreaming particles within the vacuum gap rather than particles transporting through the wedge. For the case with a cone spanning the gap (c), the effect was further enhanced, Fig. 2(f), with 10× higher peak signal and 18× higher integrated signal compared to the freestanding case, Fig. 2(g). These data indicate confinement of the proton beam in one dimension by the wedge and in two dimensions by the cone.

The proton beam spectrum from all targets was collected with the TPIE which was apertured with a pinhole in the forward direction with solid angle ~10^−7^ *sr*. Extrapolation of the full proton beam was made by multiplying the measured solid angle on layers of RCF in a separate shot day. The beam angular distribution was similar shot-to-shot. For a shot with a free hemi with no structure and no foil, the spectrum had a characteristic slope temperature of ${T}_{p}=2.7\,MeV$, maximum energy ${E}_{max}=19\,MeV$ and inferred beam energy $\varepsilon =45\,J$, or 3.7% of the laser energy for protons with $E > 3\,MeV$. The maximum energy and inferred beam energy drop somewhat for the free hemi case with a metal foil (${E}_{max}=16\,MeV$, $\varepsilon =39\,J$) and moreso when the wedge and cone structures are attached (with the lowest case being ${E}_{max}=12\,MeV$, $\varepsilon =32\,J$). Example spectra are shown in the Supplementary Information Supp. Fig. 1. The TPIE data also showed a beam of C^6+^ with nominally 15x lower signal (PSL) peaking at 15 *MeV* energy. Such carbon ions would couple effectively to the Cu foil. However, their contribution to heating would be less than from protons due to reduced numbers. Further, the heating would occur after the most intense proton isochoric heating (the fastest C ion arrives after 25 *ps*). Their contribution to Cu K*α* through C particle-induced X-ray emission (PIXE) is expected to be significantly less than from proton PIXE because the carbons have fewer particles and lower PIXE cross-section based on limited measurements in the literature at lower projectile energy in Cu^33^ and comparable projectile energy in Zn^34^.

We can estimate proton beam parameters at the Cu foil as follows. 32 *J* of protons passing through the full aperture of the cone, 200 *μm*, during a 20 *ps* window (i.e. within the time-of-flight from hemi to cone tip for all protons with >1.5 *MeV*) corresponds to 2 × 10^28^ *p*/(*cm*^2^ *s*) luminosity, 1.6 *TW* power, and 5 × 10^15^ *W*/*cm*^2^ intensity. These instantaneous values are comparable to or exceed typical peak values in conventional proton beam sources. For example, a single bunch from the Linac2 injector used at the Large Hadron Collider has peak power 1 *GW*. The average power of the laser-driven source is much lower than conventional ion beamlines.

**Figure 3:**
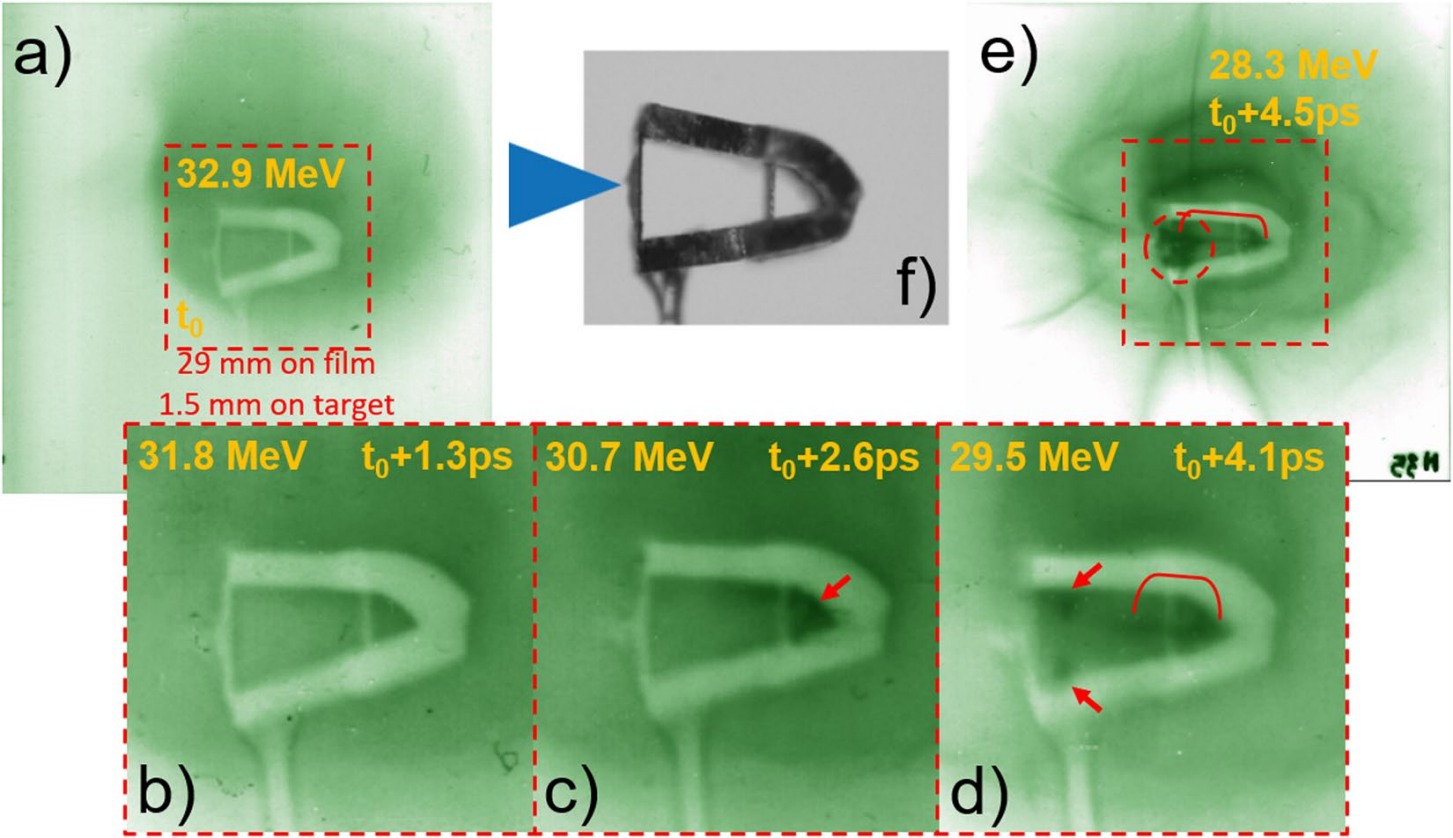
Proton radiographs showing picosecond-timescale dynamics as the main target is irradiated. (**f**) shows a photograph of a folded wedge target. A sequence of five images from the same shot are shown with relative times as indicated (**a**–**e**). Three consecutive frames (**b**–**d**) are zoomed to show excess dose (darkening) around the rear inner walls (arrows) and a band on the center plane (bracket) indicating a focussing field between the top and bottom wedge segments. By 4.5 ps into the interaction (**e**), the probe protons accumulate into a dark patch (circle). Meanwhile, the target becomes rapidly charged, driving a current in the stalk and launching expanding sheaths.

Proton radiography of the wedge case supports the focussing explanation. Five frames are shown in Fig. 3 including three consecutive, zoomed frames. The most interesting feature of the frames is a dark band that originates near the rear of the wedge on the center plane. It develops from the rear and then zips toward the curved target as seen in the sequence. This is explained by a transverse field along the wedge inner walls, directed toward the center plane. The field causes the probe protons above and below the plane to overlap on the detector forming the dark band (higher exposure) and they zip together from the back to front because the top and bottom fields are close together at the rear. This is the same field that focusses the protons from the hemi in the wedge and cone cases. Additionally, a dark patch was observed in the space behind the hemi and between the wedges. These features persist in the subsequent 21 frames (til ${t}_{0}+64\,ps$) after which the films are saturated. Deflection of probe protons was evaluated using a particle in cell pusher with prescribed fields described in the Supplementary Information.

**Figure 4:**
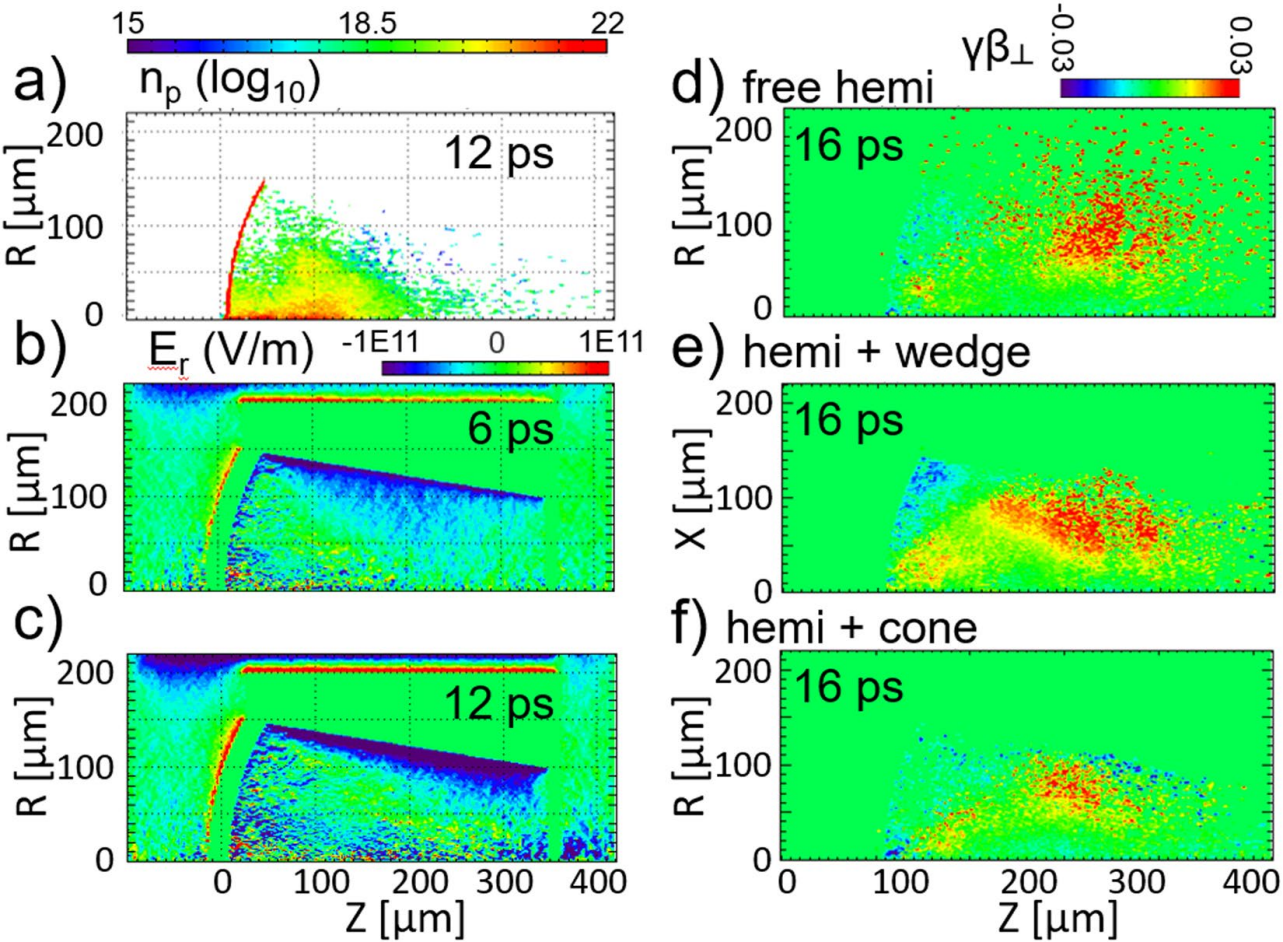
LSP simulations of proton expansion from a hemi and focussing with a rear cone structure using a source of fast electrons to represent the interaction of the OMEGA EP laser. For a hemi + cone case, (**a**) shows the proton density at time 12 *ps*; (**b**,**c**) show the transverse electric field ${E}_{r}$ at 6 *ps* and 12 *ps*, respectively. (**d**–**f**) Show the macroparticle transverse velocities for the free hemi, hemi + wedge, and hemi + cone cases, respectively.

A two step process was applied to simulate the experiment using the particle in cell code LSP in two dimensions (2D)^35^. The first step studied the accelerating and focussing dynamics. Three cases are presented: the cone freestanding target case simulated and the conical target case using 2D cylindrical coordinate systems, and the wedge case in a 2D cartesian system. The configuration details are given in the Methods Section. In the freestanding case, protons are initially focussed due to the curvature of the target. However, a significant fraction of them diverge away Fig. 4(d). In the wedge and cone cases, a strong focussing field is observed to persist along the inner cone surface. For the cone case the radial focussing field strength (seen during and after the laser in Fig. 4(b,c) is comparable to the initial field from the curved target, confining protons Fig. 4(b), near the hemi/cone conjunction). Figure 4(d–f) show macroparticle velocities 16 *ps* into the simulations for the three cases. For the free hemi case, it is clear that the beam is already diverging at $Z=200\,\mu m$, even before the nominal focal position for the curved target, and many particles are more lateral than the radius of the hemi. For the wedge case, the walls of the wedge are a physical barrier to protons. For the cone case, particles can be seen to reflect toward the central axis, corralling and collimating the proton beam.

We can again estimate beam parameters by checking the numbers in the simulations. By integrating the profile of the beam at the back of the cone, we find that 5 × 10^13^ protons passed through a disk-shaped plane of 100 *μm* diameter in 41 *ps* with an average energy of 1.2 *MeV*. Thus, the beam density averaged across the cone hole and averaged during this time is $5\times {10}^{27}\,p/(c{m}^{2}s)$ and the intensity is $7\times {10}^{14}\,W/c{m}^{2}$. During a $20\,ps$ window (96% of the protons reaching the extraction plane arrived during 21–41 *ps*) and looking inside 30 *μm* radius, the beam density is $3\times {10}^{28}\,p/(c{m}^{2}s)$. The highest intensity is $1\times {10}^{16}\,W/c{m}^{2}$. These values are in near agreement to the estimates based on the TPIE detailed above. We note that at the end of the 41 *ps* simulation many slow particles that carry significant energy but that do not contribute to the most intense phase of the interaction have not reached the extraction plane.

**Figure 5:**
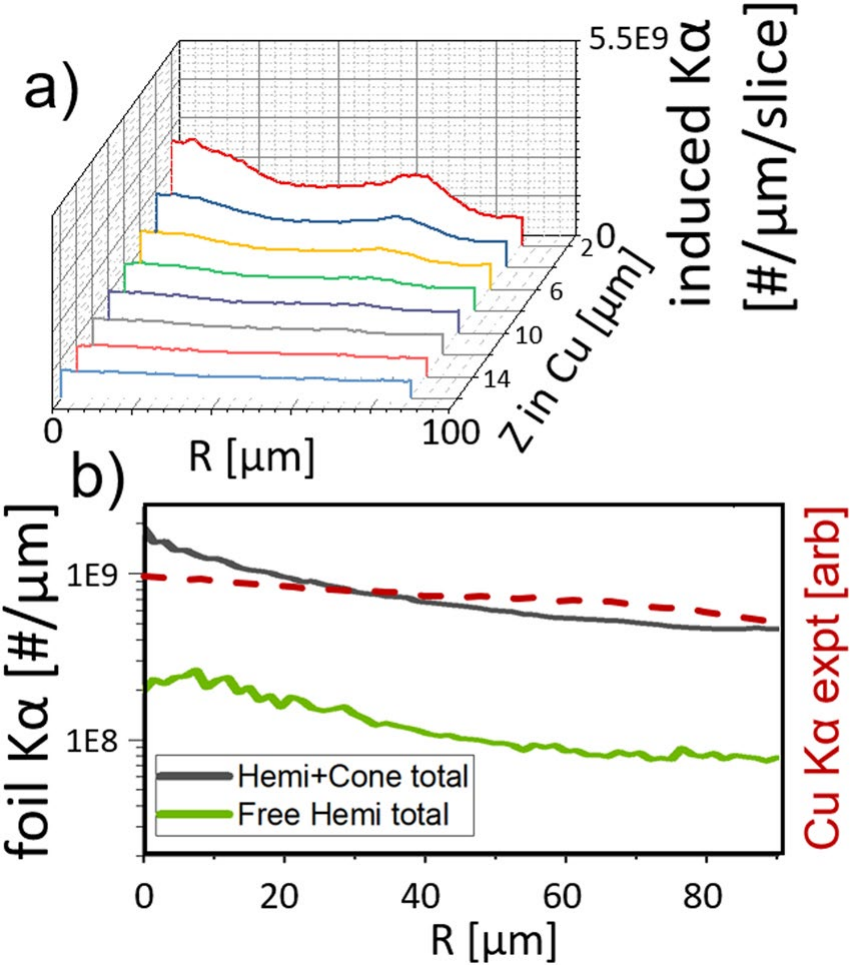
K*α* post-processing in the Cu transport simulation. (**a**) lateral profiles of proton-induced Cu *Kα* photons at 2 *μm* intervals in a hemi with wedge case. (**b**) composite lateral profile for a 10 *μm* foil for the free hemi case and hemi with cone case. An experimental lineout of the hemi-cone case is overlaid.

In the second modeling step, deposition and induced *Kα* photons in the rear Cu layer are studied. In each of the three cases above, the electrons and protons that made it to the rear foil were recorded and injected into a Cu slab (see Methods for details). The time-integrated Cu K*α* binned by lateral position and depth into the foil is shown in Fig. 5(a). The photons can be traced back to three distinct populations of particles. Hot electrons, protons, and quasi-neutralizing electrons that co-move with the protons. Hot electrons moving through the cone contribute a non-negligible but uniform K*α* signal while the latter two produce a centrally peaked Cu K*α* signal. Figure 5(b) shows that the hemi with cone results in 10× higher peak Cu K*α* signal than the free hemi case, in accordance with the experimental measurement.

**Figure 6:**
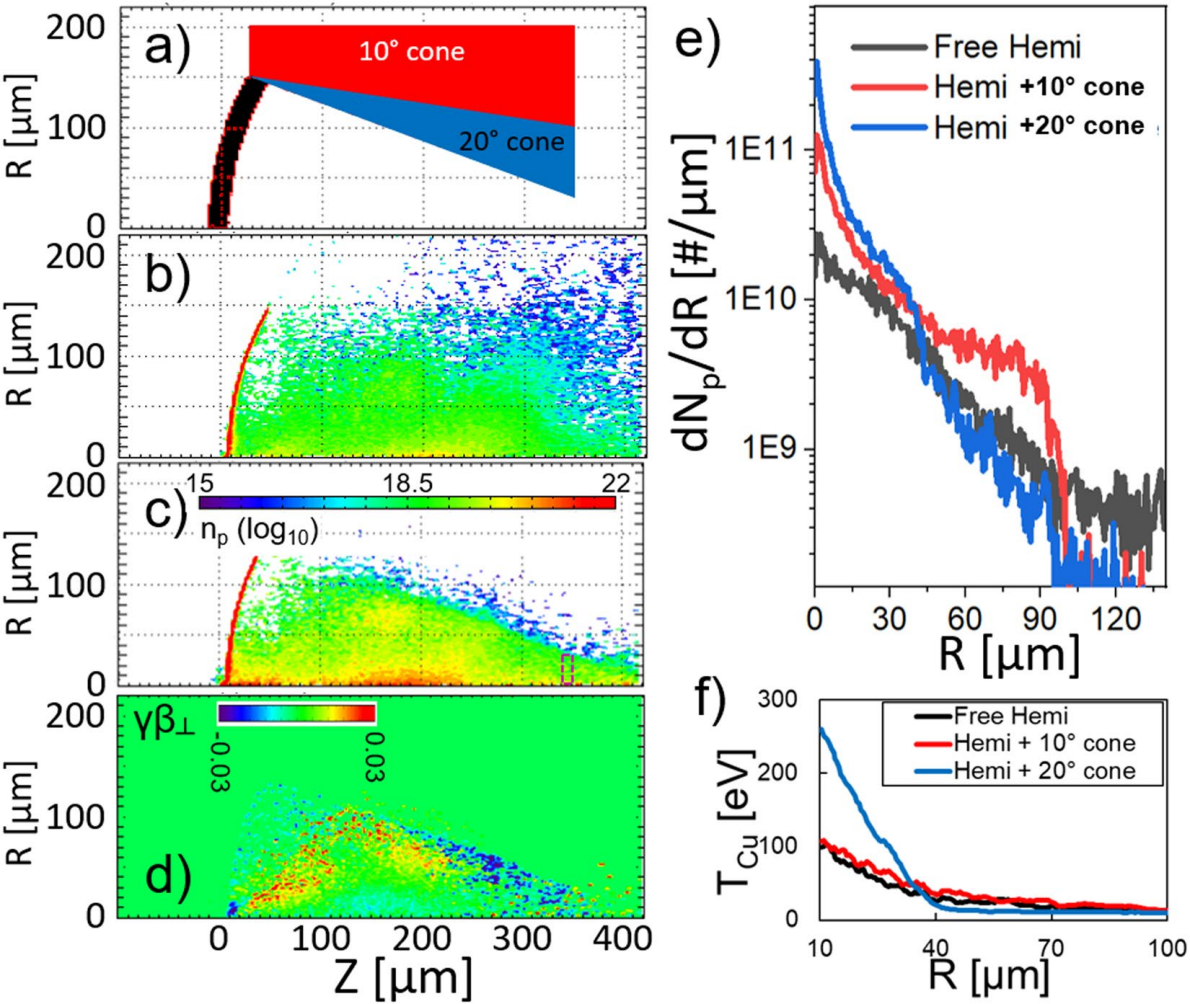
(**a**) simulated target geometry for the standard cone and 20 deg cone. (**b**) proton density for the free hemi at 22 *ps*. (**c**) proton density for the 20 deg cone at 20 *ps*. (**d**) radial velocity of proton macroparticles for the 20 deg cone at 16 *ps*. (**e**) the radial distribution of protons reaching the purple box shown in (**c**) through 33 *ps*. (**f**) Cu ion temperature radial profile for the same three cases.

As an additional test of the focussing possibilities, a radial simulation was carried out with a steeper cone angle of 20° as portrayed in Fig. 6(a). Comparing Fig. 6(b,c), it can be seen that the proton beam is significantly stronger on axis for the steep cone case than the free hemi. Figure 6(d) shows that the majority of proton macroparticles are reflected toward the axis near the cone tip as compared to the other cases in Fig. 4(d–f). Figure 6(e) shows that the standard cone focusses 5× the number of particles to the axis as the free hemi, while the steep cone would produce even tighter focussing. In the Cu transport simulations it can be seen how the proximal target structures enhance heating of a sample. Figure 6(f) shows the radial temperature profile in the Cu mid-plane for the three cases. The hemi with standard cone heats to a 15–20% higher temperature than the free hemi would for radii out to 100 *μm*, while the 20° cone heats to at least twice the nominal temperature for radii out to 40 *μm*. The temperature difference between the free hemi and standard cone case at this position is not as striking as the particle distribution 6(e) nor the Cu *Kα* data because the latter two are weighted to the numerous protons entering the Cu with 10–1000 keV that do not penetrate to the Cu mid-plane.

## Conclusion

We have shown that the proton beam from a $1.25\,kJ$, $\tau =10\,ps$ laser could be focussed effectively using a conical structure. Direct evidence of focussing is seen in imaged *Kα* emission from a Cu foil placed at the end of the cone. For the case with a structured target the total emission of Cu K*α* fluorescence was increased by a factor of $18$ in experiment. Simulations show that electrostatic, persistent fields on the cone inner wall funnel protons to the cone tip and that the *Kα* signal increase is a combination of extra signal due to electrons directly moving through the target but also a significant constriction of protons into a collimated spot of roughly the cone tip size. We note that the simulation provided valuable identification of the beam behaviors, but there may be quantitative disagreements due to the 2D description especially for the hemi-wedge case. The 10 *ps* pulse duration is critical to the focussing effect since the beam of protons takes 10 s of ps to transit the focussing structure. We presented important proton acceleration data in a laser parameter range that has barely been explored and is much more relevant to proton FI than the majority of laser-driven proton studies using freestanding foil targets and sub-picosecond lasers. Based on the measurements the proton intensity is >1% of the focussed laser intensity, an important figure of merit for proton FI, and the beam current density at focus is likely high enough to induce modified stopping behaviors predicted by modeling^36^. The simulated steeper cone produced further focussing and heated the Cu foil to >150 *eV* in the central 40 *μm* diameter. Further, we hypothesize that a thin-walled cone would be an even more effective lens and retain the benefits of a reduced mass target.

## Methods

### Experimental configuration

The experiment was conducted with orthogonal short pulse beams from OMEGA EP. The main beam ($1250\,J,\,10\,ps,\,35\,\mu m\,{r}_{80}$, where *r*_80_ is the radius containing 80% of the spot energy) was incident on the target apex in line with the axis of symmetry of the spherical cap and the cone. A 3-dimensional representation of the target and important diagnostic lines of sight is shown in Fig. 2. The Spherical Crystal Imager (SCI^37^) fielded a spherical quartz crystal from 63 deg above the equatorial plane, directly above the cone axis. The foil stacks were $600\times 300\,\mu m$ and positioned either 300 or 450 *μm* behind the planar portion of the hemi target. When present, the cone or wedge structure was directly glued to the hemi and the foil stack. The rear layer was a 10 *μm* thick Cu foil; it was either uncoated or coated with a front layer of 13 *μm* Al or 6 *μm* Ag, the importance of which is beyond the scope of this paper. There were no measurable differences observed in the *Kα* from targets with Al vs Ag. Their thicknesses were chosen to have the same stopping range for protons. The beam used for proton radiography ($850\,J,\,10\,ps,\,20\,\mu m\,{r}_{80}$) was incident normally on a 1 *mm* square 10 *μm* thick Au foil. A custom arrangement of radiochromic films was used with 100 *μm* Al foil in front, 53 slices of film, and minimal filters between, to preserve a high degree of temporal discrimination.

### Particle-in-cell simulations of particle generation, transport, and induced Cu K*α*

**Figure 7:**
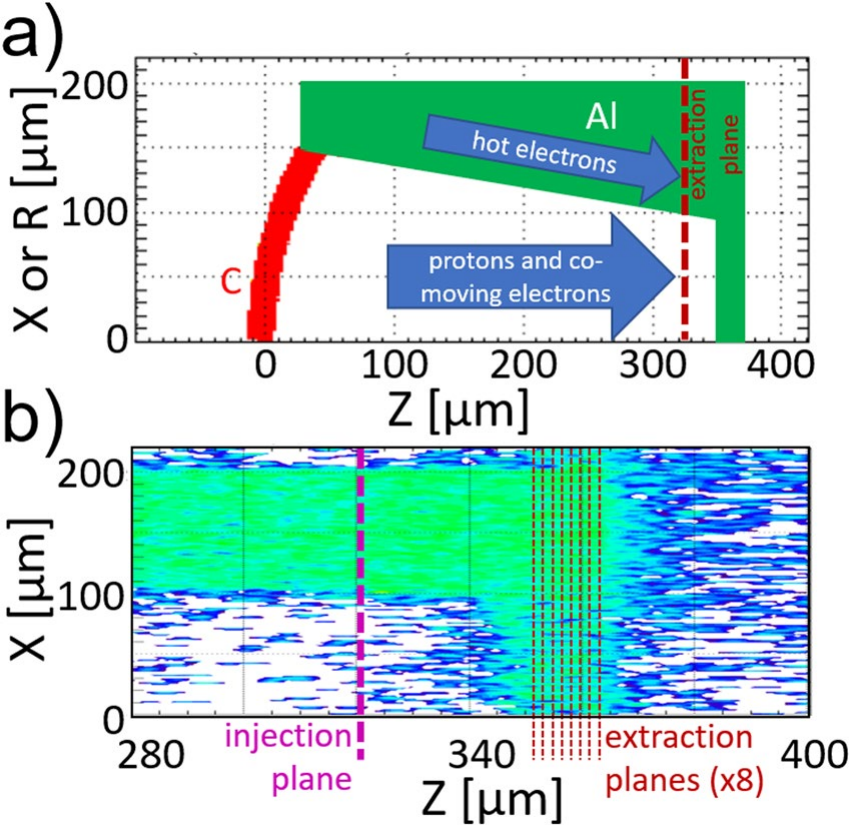
(**a**) The simulation geometry showing one half of the domain for a simulation in cartesian cordinates (X) or the whole domain for cylindrical coordinates (R). (**b**) Closeup of the Cu foil in a cartesian run, showing where hot electrons and protons are counted in the middle of the Cu region. The plotted quantity is electron density with the same color scale as Figs. 4(a) and 6(b,c).

Particle-in-cell code LSP was used to study kinetic aspects of the main target. The hemi + wedge target geometry was represented including a preplasma, C curved foil, Al trapezoidal sidewalls and rear foil. For simplicity, the rear foil was modeled as Al for the first simulation. The geometry is shown in Fig. 7(a). By using LSP’s implicit push algorithm and the fluid description in the solid regions of the target and Al structures, the number of macroparticles/cell limits could be significantly relaxed comparing to typical PIC simulations. In these fluid regions, particles follow the same equation of motion of kinetic particles but a collision term using pressure and frictional forces is added. Meanwhile, the particles that are responsible for the TNSA effect (namely hot electrons and surface protons) are treated kinetically with high particle density, fully resolving the acceleration. In our simulations, 4 particles/cell for each species were laid out in the solid regions and 625 particles/cell filled the 1 *μm* thick hydrogen layer of the Hemi target. The minimum grid size was 0.4 *μm* for non-uniform grids. Simulations outputted the snap shots of fields and particle densities every 0.5 *ps*. Data was accumulated on extraction planes every time step, 0.4 *fs*.

To make the problem tractable with finite computational resources, the laser-target interaction process was bypassed- a population of electrons was injected to emulate the laser interaction. The electron source had total energy 30% of $1250\,J=375\,J$ in 20 *ps* total duration with $10\,ps\,FW\,HM$ Gaussian history and $35\,\mu m\,FW\,HM$ Gaussian spatial profile. The energy spectrum had slope temperature linearly increasing to 3 *MeV* until the laser peak and a 30% Gaussian energy spread in a transverse direction. The electron source method has been applied previously in similar problems^30^ and benchmarked against full simulation of the laser^31^. The choice of an electron source with increasing slope temperature that exceeds that predicted by ponderomotive scaling for the laser intensity is a proven method for more accurately simulating proton acceleration from multi-picosecond pulses^38^. Longitudinal and lateral electric field maps were recorded periodically as were all species’ densities, temperatures and velocities.

Electrons reached the rear foil primarily (in number) by traveling within the target structure and along the walls while protons drift through the gap. All particles, including protons, hot electrons, and co-moving electrons, that nearly reached the rear foil ($Z=325\,\mu m$) were recorded in the first simulation then input into a separate transport simulation at the recorded position in front of a Cu slab, preserving each particle’s momentum, lateral position, and relative timing.

In the Cu transport simulations, the injection plane was set before the entrance of the transport layer (Cu slab) to enable multiple transport simulations without having to re-simulate the long time of flight of particles from the hemispherical target. The domain is shown in Fig. 7(b). The particles collected in the longer simulation were re-injected in the Cu transport simulation with the same temporal and spatial profiles as collected and at the same plane along the cone. The collection/injection plane was chosen in front of the Cu slab to avoid field effects at the slab boundary. A vacuum gap was included in the domain so that fields could evolve in time without numerical disturbance.

A series of extraction planes recorded the particles’ momenta and positions every 2 *μm* into the slab. Particle stopping calculations in the slab applied Atzeni’s approach for relativistic electrons^39^ and a bound plus free electronic stopping model for protons^40^. The PIXE cross-sections for electron- and proton-induced Cu $K\alpha$ emission^41,42^ are applied with the simplifying assumption that the particle energies are constant over each 2 *μm* slice. The shape of the Cu kα emission profile can be plotted using the particle positions, and the cumulative profile that would be observed from the rear of the foil is then calculated by adding the slices, accounting for x-ray opacity.

## Supplementary information

Supplementary Information
