# Supplementary material for: Focussing Protons from a Kilojoule Laser for Intense Beam Heating using Proximal Target Structures
Source: Sci Rep. 2020 Jun 10;10:9415. doi: 10.1038/s41598-020-65554-4 (PMC7287069; doi:10.1038/s41598-020-65554-4)
Supplement: Supplementary file 1 — Supplementary Information [file 41598_2020_65554_MOESM1_ESM.pdf]

Focussing Protons from a Kilojoule Laser for Intense Beam Heating using Proximal Target Structures

C. McGuffey[1], J. Kim[1], M.S. Wei[2], P.M. Nilson[3], S.N. Chen[4,6], J. Fuchs[4], P. Fitzsimmons[2], M.E. Foord[5], D. Mariscal[1,5], H.S. McLean[5], P.K. Patel[5], R.B. Stephens[2], F.N. Beg[1]

[1] *Center for Energy Research, University of California, San Diego, La Jolla, CA 92093-0417, USA*
[2] *General Atomics, P.O. Box 85608, San Diego, CA 92186-5608, USA*
[3] *Laboratory for Laser Energetics, University of Rochester, Rochester, NY 14623, USA*
[4] *Laboratoire pour l'Utilisation des Lasers Intenses - CNRS, CEA, UPMC Univ Paris 06 : Sorbonne Universit, Ecole Polytechnique, Institut Polytechnique de Paris - F-91128 Palaiseau cedex, France*
[5] *Lawrence Livermore National Laboratory, P.O. Box 808, Livermore, CA 94550, USA and*
[6] *Extreme Light Infrastructure - Nuclear Physics / Horia Hulubei National Institute for R&D in Physics and Nuclear Engineering, Bucharest-Magurele, 077125, Romania*

(Dated: April 7, 2020)

I Example proton spectra

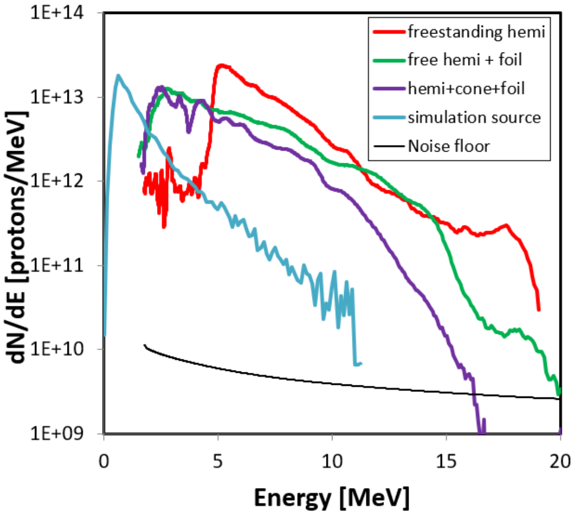

SUPP.FIG. 1: Proton spectra measured for three target types with the TPIE diagnostic.

II Proton radiography tracking code

In order to ascertain whether the predicted fields surrounding the target could account for the features observed in the proton radiography data, the LSP particle in cell code was used to track protons through a prescribed field topology. A three-dimensional 1 mm cube domain was created. Two-dimensional electric field data from the LSP hemi + wedge transport simulation (analogous to Fig. 3(b) or (c) but in cartesian coordinates) was extruded uniformly through 300 μm depth in the probing axis, equal to the wedge breadth on the experimental target. Examples of the total electric field, $\sqrt{E_x^2 + E_z^2}$, for two different snapshots into the transport simulation are shown in SUPP.FIG. 2(left). A 1x1 mm sheet of protons with kinetic energy $28.1 - 28.5\ MeV$, corresponding to the Bragg absorption band from a frame in Fig. 2, was sent from a virtual point in front of the domain. Protons reaching the back of the domain were then treated ballistically for an 80 mm transit to the radiochromic film position. Maps of the proton density are shown as simulated radiographs in SUPP.FIG. 2(right).

Probe protons deflect significantly as they pass through the target vicinity due to the electric fields, and significant bunching is observed at the simulated radiograph plane. Features of interest are marked with the same annotation marks as in Fig. 2(c),(d) and (e). Dark features are consistently seen in regions where the total electric field is low or where the fields directions converge such as at the hemi/wedge interface. While the absolute timings do not match with the experimental values, which may be due to the subtlety of the true experimental pulse rise, the simulated radiographs and data show a similar development over just a 2 ps window.

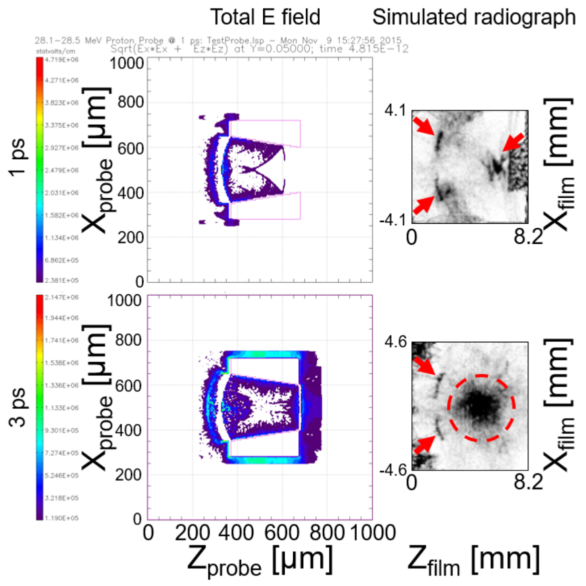

SUPP.FIG. 2: (left) field maps of composite electric field strength for 1 *ps* and 3 *ps* (right) simulated radiographs for the same times
